# Computational Insights into the Polypharmacological Landscape of BCR-ABL Inhibitors: Emphasis on Imatinib and Nilotinib

**DOI:** 10.3390/ph18070936

**Published:** 2025-06-20

**Authors:** Rima Hajjo, Dima A. Sabbah, Raghad Alhaded, Aye Alquabe’h, Sanaa K. Bardaweel

**Affiliations:** 1 Department of Pharmacy, Faculty of Pharmacy, Al-Zaytoonah University of Jordan, Amman 11733, Jordan; dima.sabbah@zuj.edu.jo (D.A.S.); raghadhdd2@gmail.com (R.A.); ayajamal048@gmail.com (A.A.); 2 Laboratory for Molecular Modeling, Division of Chemical Biology and Medicinal Chemistry, Eshelman School of Pharmacy, The University of North Carolina at Chapel Hill, Chapel Hill, NC 27599, USA; 3 Department of Pharmaceutical Sciences, School of Pharmacy, University of Jordan, Amman 11942, Jordan; s.bardaweel@ju.edu.jo

**Keywords:** BCR-ABL inhibitors, drug repurposing, imatinib, kinase inhibitors, nilotinib, pharmacoinformatics, polypharmacology

## Abstract

**Background:** BCR-ABL inhibitors such as imatinib and nilotinib exhibit multi-kinase activity that extends beyond oncology, offering significant potential for drug repurposing. **Objectives:** This study aims to systematically evaluate and prioritize the repurposing potential of BCR-ABL inhibitors, particularly imatinib and nilotinib. **Methods:** An integrated pharmacoinformatics framework was applied to analyze seven BCR-ABL inhibitors. Structural clustering, cheminformatics analysis, and transcriptomic profiling using the Connectivity Map were employed to evaluate structural relationships, target profiles, and gene expression signatures associated with non-oncology indications. **Results:** Structurally, imatinib and nilotinib clustered closely, while HY-11007 exhibited distinct features. Nilotinib’s high selectivity correlated with strong transcriptional effects in neurodegeneration-related pathways (e.g., HSP90 and LYN), whereas imatinib’s broader kinase profile (PDGFR and c-KIT) was linked to fibrosis and metabolic regulation. Connectivity Map analysis identified more than 30 non-cancer indications, including known off-target uses (e.g., imatinib for pulmonary hypertension) and novel hypotheses (e.g., nilotinib for Alzheimer’s via HSPA5 modulation). A substantial portion of these predictions aligned with the existing literature, underscoring the translational relevance of the approach. **Conclusions:** These findings highlight the importance of integrating structure–activity relationships and transcriptomic signatures to guide rational repurposing. We propose prioritizing nilotinib for CNS disorders and imatinib for systemic fibrotic diseases, supporting their advancement into preclinical and clinical evaluation. More broadly, this framework offers a versatile platform for uncovering hidden therapeutic potential across other drug classes with complex polypharmacology.

## 1. Introduction

Breakpoint Cluster Region–Abelson Murine Leukemia Viral Oncogene Fusion Protein (BCR-ABL) inhibitors, such as imatinib, nilotinib, and related compounds, were originally developed for the treatment of chronic myeloid leukemia (CML) and other cancers expressing the BCR-ABL fusion oncogene [1]. More recently, these inhibitors have demonstrated promising potential in non-cancer indications, owing to their multi-kinase activity and off-target inhibition of kinases such as PDGFR, c-KIT, FLT3, and native ABL1 [2]. Moreover, their adverse effects, including cardiovascular toxicity, fluid retention, and cytopenias, further highlight their polypharmacological profiles [3]. Clinical investigations have also explored the use of nilotinib and bosutinib in neurodegenerative diseases, providing further evidence of their therapeutic versatility beyond oncology [4].

Imatinib (Gleevec®) was the first tyrosine kinase inhibitor (TKI) designed to selectively inhibit the BCR-ABL fusion protein, which results from the oncogenic Philadelphia chromosome translocation occurring in chronic myeloid leukemia (CML) and some cases of acute lymphoblastic leukemia (ALL) [5]. It has moderate selectivity towards BCR-ABL. It also targets ABL1, ARG, c-KIT (CD117), DDR1, NQO2, PDGFRα, and PDGFRβ. Nilotinib is a highly selective BCR-ABL inhibitor with minimal off-target kinase activity compared to imatinib and other BCR-ABL inhibitors targeting the ATP site, but it is less selective than asciminib (ABL001), which binds allosterically to the myristoyl pocket of ABL1 [6]. Imatinib and nilotinib bind the ATP-binding site of BCR-ABL; both bind the inactive form of the ABL protein.

Despite their primary use in oncology, BCR-ABL inhibitors have shown emerging promise in non-cancer indications, largely attributable to their inhibition of kinases implicated in fibrosis, neurodegeneration, immune modulation, and metabolic regulation [7]. Therefore, BCR-ABL inhibitors are not only historically significant for their pioneering role in targeted cancer therapy via well-characterized mechanisms, but are also pharmacologically versatile, making them unique tools for both precision oncology and the expanding field of drug repurposing and systems pharmacology. This expanding therapeutic scope is supported not only by preclinical studies but also by the clinical observations of their off-target activities. Understanding the structural determinants, transcriptional effects, and network pharmacology underlying the broader impact of BCR-ABL inhibitors is therefore critical for rational drug repurposing and precision medicine initiatives.

In this study, we present a comprehensive informatics workflow to explore the structural, biological, and therapeutic diversity of BCR-ABL inhibitors, with a particular focus on the distinct transcriptomic profiles of imatinib and nilotinib. We begin by applying cheminformatics approaches to evaluate the chemical and structural diversity across the BCR-ABL inhibitor class. Subsequently, we employ a chemogenomics strategy to characterize the transcriptional effects of these compounds and generate gene expression signatures. These signatures are then used to query the Connectivity Map (CMap) [8] database, the gene expression L1000 platform version 1.0, to identify compounds and genetic perturbations that elicit similar gene expression responses to BCR-ABL inhibitors. Finally, the matched compounds and gene perturbations are prioritized and examined to generate polypharmacology hypotheses, which are further supported through evidence mined from the biomedical literature. This integrative and cost-effective strategy provides a scalable tool for investigating the polypharmacology of other drug classes and guiding hypothesis-driven drug repurposing projects.

## 2. Results

**Figure 1:**
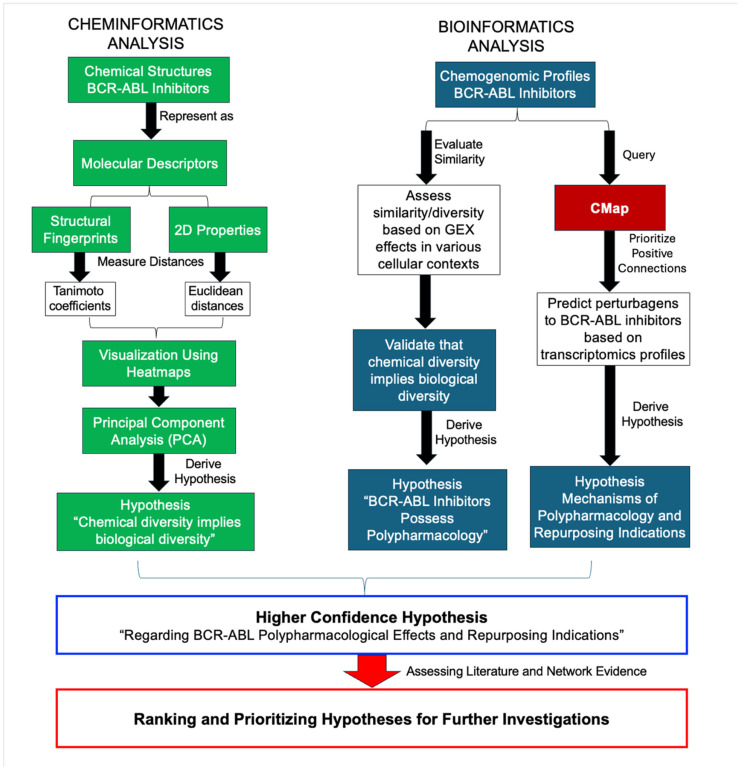
Integrative informatics workflow to study the polypharmacology of BCR-ABL inhibitors.

An informatics workflow (Figure 1) was developed and applied to study the network pharmacology of BCR-ABL inhibitors, based on methods developed by Hajjo et al. [9,10,11,12], to formulate testable hypotheses regarding non-cancer indications and polypharmacological mechanisms.

### 2.1. Chemical Dissimilarity

**Figure 2:**
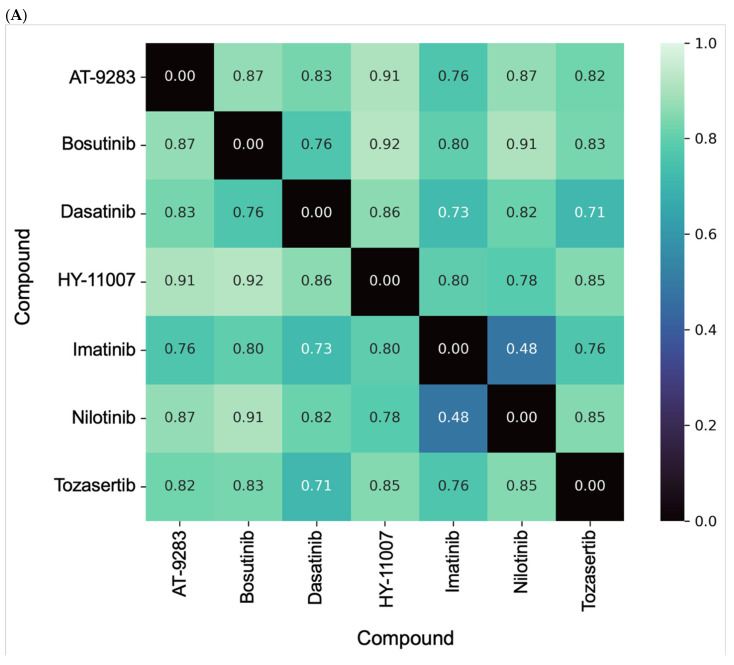

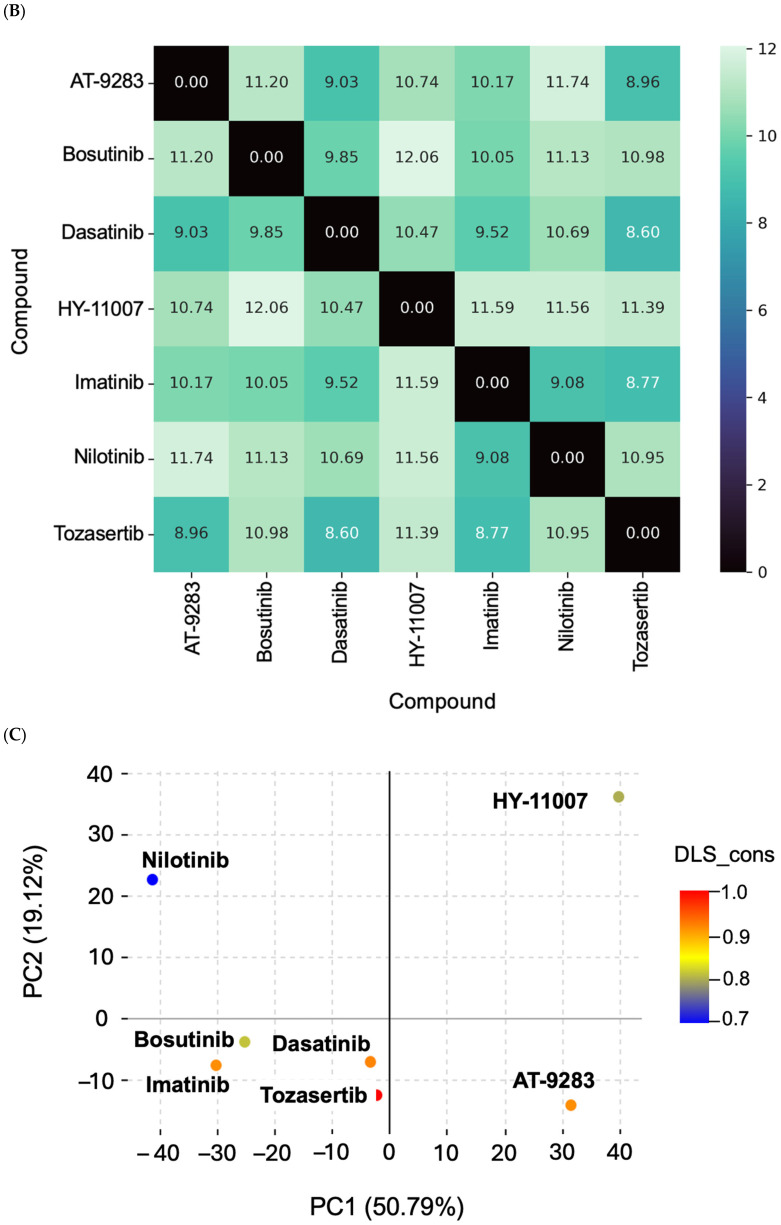
Cheminformatics analysis of BCR-ABL inhibitors. (**A**) Heatmap for the distance matrix using Tanimoto coefficients and Morgan fingerprints. (**B**) Heatmap for the distance matrix using Euclidean distances and 2D alvaDesc molecular descriptors. (**C**) Principal component analysis of seven BCR-ABL inhibitors using 2D alvaDesc molecular descriptors and Euclidean distances. The color gradient reflects DLS_cons—the consensus drug-likeness score—providing an additional interpretive layer grounded in the pharmacokinetic and physicochemical profile of the molecules. The plot reveals the principal component scores for PC1 (50.79%) and PC2 (19.12%), which together explain ~70% of the total variance, suggesting good dimensional reduction and data representation fidelity.

All seven BCR-ABL inhibitors analyzed contained heterocyclic cores (e.g., pyrimidine, quinoline, and thiazole), which is essential for ATP-binding site interactions; this is a feature that is linked to cross-pharmacology with kinases like SRC and aurora kinases. Cheminformatics analysis using pairwise Tanimoto distances from binary fingerprints (Figure 2A) showed that nilotinib and imatinib were the most similar (distance = 0.48), while bosutinib, dasatinib, and tozasertib formed a moderately similar subcluster (~0.7–0.8); HY-11007 and AT-9283 were more structurally distinct (>0.85). Pairwise Euclidean distances and 2D alvaDesc descriptors (Figure 2B) captured physicochemical dissimilarity, with tozasertib–dasatinib and tozasertib–imatinib showing the greatest similarity (distances ~8.6–8.8), and nilotinib and HY-11007 showing the greatest divergence (≥11.5). This broader range (~8.6 to 12.1) indicated that Euclidean metrics detected subtler variations compared to Tanimoto distances. A total of 3874 2D molecular descriptors (Supplementary Table S1) were calculated out of 5305 available descriptors, after excluding all 3D descriptors. Of these, 1889 descriptors were used for the principal component analysis (PCA) following the elimination of descriptors with constant or near-constant values, descriptors with at least one missing value (or all missing values), and descriptors with a standard deviation less than 0.0001.

The principal component analysis (PCA) of the descriptors (Figure 2C) further confirmed chemical diversity, i.e., nilotinib and HY-11007 were well-separated along the *Y*-axis, while imatinib, bosutinib, dasatinib, and tozasertib clustered together. AT-9283 appeared isolated along the *X*-axis, reflecting unique physicochemical features. The DLS_cons color scale (red = high consensus similarity) visualized relative drug-likeness. Overall, complementary molecular representations effectively revealed structural and physicochemical diversity among the inhibitors.

### 2.2. Biological Dissimilarity

**Figure 3:**
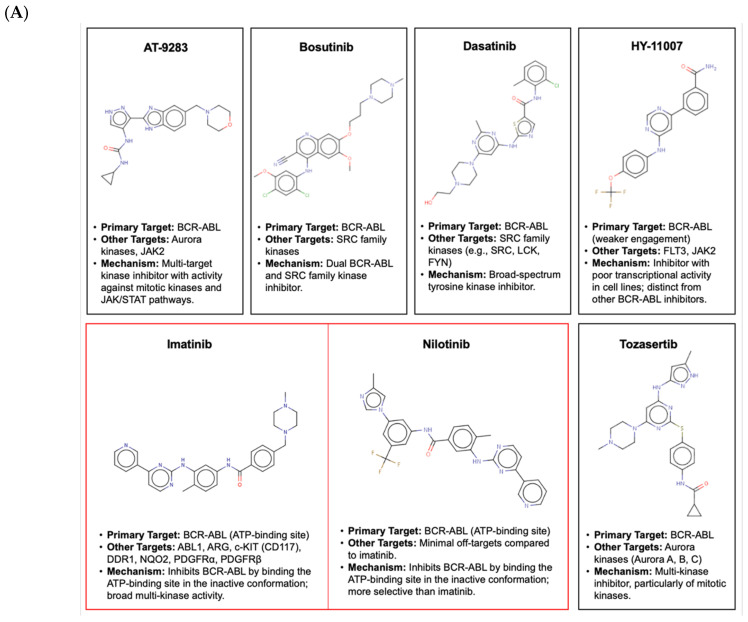

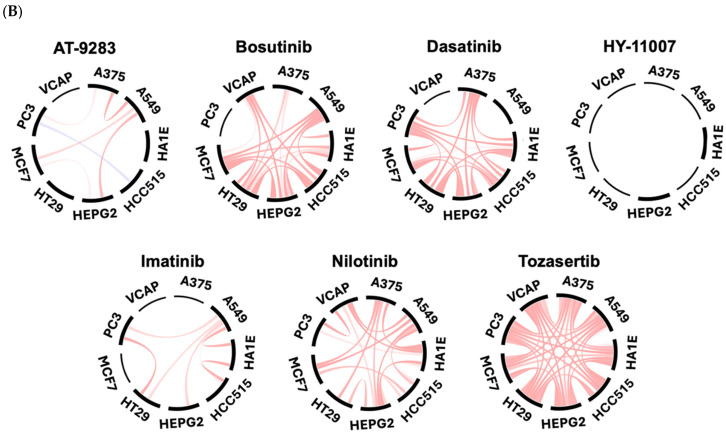
Structural and transcriptional signatures underlying the polypharmacology of BCR-ABL inhibitors. (**A**) Chemical structures, target profiles, and mechanisms of action of BCR-ABL inhibitors. (**B**) Cell-line-specific transcriptional responses to BCR-ABL inhibitors. Thick black bars signify transcriptional activity scores (TASs) greater than or equal to 0.5; thinner black bars indicate scores less than 0.5. Red lines (chords) denote similar positive connectivity scores between cell lines, which range from 80 to 100 (pale to intense color according to the score). Chords are shown only when TASs are ≥0.5, indicating strong or biologically relevant transcriptional activity. The absence of red chords either means that the compound’s TAS is very low, or that no data are available. Source: CMap database.

The seven BCR-ABL inhibitors analyzed, identified from the Connectivity Map (CMap) based on their annotated mechanisms of action (Figure 3A), exhibited distinct pharmacological profiles. Evidence from the literature ranks nilotinib as the most selective BCR-ABL inhibitor, followed by imatinib. AT-9283 and tozasertib also target aurora kinases, while bosutinib and dasatinib more potently inhibit SRC family kinases. HY-11007, in contrast, primarily targets FLT3 and JAK2, distinguishing it mechanistically. CMap data further supported these differences at the transcriptional level. As shown in Figure 3B, the inhibitors displayed diverse transcriptional activity scores (TASs) and connectivity patterns across nine cancer cell lines. Tozasertib, dasatinib, and nilotinib induced strong, consistent responses (TAS ≥ 0.5), reflected by thick black bars and dense red connectivity chords, indicating broad-spectrum activity. In contrast, imatinib and AT-9283 showed moderate, cell-line-specific responses, while HY-11007 exhibited minimal transcriptional activity, suggesting limited cellular engagement despite structural similarity to the other inhibitors.

### 2.3. Hypothesis Generation Polypharmacological Effects

**Figure 4:**
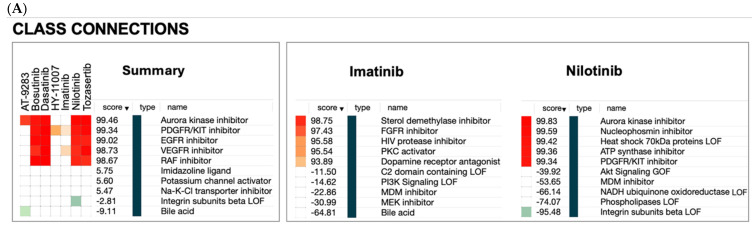

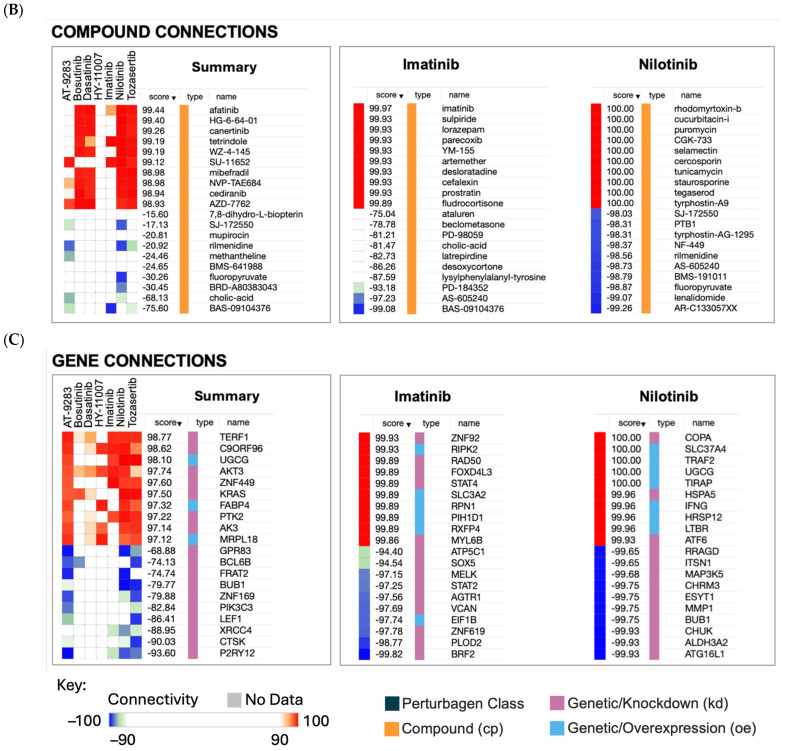
CMap connections with BCR-ABL inhibitors across perturbagen class, compound, and genes. (**A**) Connections with seven BCR-ABL inhibitors. (**B**) Connections with imatinib. (**C**) Connections with nilotinib.

Results from the CMap analysis (Figure 4) suggest that BCR-ABL inhibitors exert broader effects beyond ABL inhibition, consistent with their profiles as multi-target kinase inhibitors. Predicted compound and genetic connections could support the generation of strong hypotheses regarding mechanisms of action, off-target effects, and drug repurposing opportunities by leveraging transcriptomic similarities identified through high-scoring positive CMap connections.

#### 2.3.1. Non-Cancer Indications of BCR-ABL Inhibitors

The CMap analysis revealed distinct transcriptional profiles among the seven BCR-ABL inhibitors (Figure 4A). Compounds such as dasatinib, bosutinib, tozasertib, and AT-9283 exhibited widespread positive and negative connections across multiple classes, compounds, and genes. A strong connectivity with unrelated perturbagen classes (e.g., aurora kinases, EGFR inhibitors, VEGFR inhibitors, PDGFR inhibitors, and HIV protease inhibitors), which are not directly tied to BCR-ABL signaling, indicated a broad polypharmacology and a reduced selectivity of inhibitors, consistent with their known multi-kinase activity.

Compound connections shown in Table 1 further confirmed strong similarities to EGFR inhibitors (e.g., afatinib, HG-6-64-01, and canertinib) and other multi-kinase inhibitors (e.g., tetrindole, SU-11652, and WZ-4-145). Negative compound connections, such as cholic acid, BRD-A80383043, and BAS-09104376, further suggested opposing transcriptional effects, possibly linked to metabolic or unknown pathways.

Gene-level connections showed strong similarity with ERF1, C9ORF96, UGC, and others, with a notable overlap with the PI3K/AKT and RAS signaling pathways (e.g., AKT3, KRAS, and FBP4), reinforcing the involvement of oncogenic signaling. Few negative gene connections (e.g., PIK3C3, XRCC4, CTSK, and P2RY12) pointed to possible effects on autophagy, DNA repair, and inflammation.

#### 2.3.2. Non-Cancer Indications of Imatinib

Imatinib connected strongly to other tyrosine kinase inhibitors, but also showed connectivity to the diverse drug classes shown in Table 2, including serotonin and dopamine receptor antagonists, indicating off-target transcriptional effects. Gene connectivity revealed a partial overlap with relevant signaling components to BCR-ABL signaling effects but also included non-BCR-ABL-related genes, suggesting a less-focused transcriptional footprint than nilotinib. The non-cancer indications connected to imatinib via CMap positive-scoring connections are diverse, covering neurological, cardiovascular, metabolic, inflammatory, and infectious diseases. There is a strong neuropsychiatric signal, broader than seen with nilotinib (Table 3), but also a clear footprint in immune modulation and vascular pathology.

**Table 2:** Top CMap positive-scoring perturbagen connections to imatinib and their known non-cancer indications.

#	Perturbagen	Type	Score	Non-Cancer Indications
1	Mineralocorticoid Agonist	Class	98.22	Orthostatic Hypotension (FDA-approved); Addison Disease (FDA-approved).
2	PLK Inhibitor	Class	97.63	Inflammation (Preclinical) [58]; HIV Infections (Preclinical) [59].
3	Dopamine Receptor Grp1	Class	96.93	Parkinson’s Disease (Clinical) [60]; Depressive Disorder (Preclinical) [61].
4	PKC Activator	Class	95.54	Alzheimer’s Disease (Clinical) [62]; HIV infection (Clinical) [62].
5	VEGFR Inhibitor	Class	95.45	Uveitis (FDA-approved); Atherosclerosis (Preclinical) [63].
6	Opioid Receptor Agonist	Class	95.09	Pain (FDA-approved); Cough (FDA-approved).
7	Dopamine Receptor Grp2	Class	92.62	Schizophrenia (FDA-approved); Parkinson’s Disease (Clinical) [60]; Alzheimer’s Disease (Clinical) [64].
8	ROCK Inhibitor	Class	90.26	Glaucoma (FDA-approved) [65]; Hypertension, Pulmonary (Clinical) [66]; Parkinson’s Disease (Clinical) [67].
9	Na-K-Cl Transporter Inhibitor	Class	89.83	Kidney Diseases; Hypertension (FDA-approved for non-selective inhibitors); Epilepsy (Clinical for selective inhibitors) [68]; Autistic Disorder (Clinical for selective inhibitors) [69].
10	Phosphodiesterase Inhibitor	Class	89.61	Erectile dysfunction (FDA-approved); Pulmonary Disease, Chronic Obstructive (FDA-approved); Heart Diseases (FDA-approved); Schizophrenia (Clinical) [70].
11	Imatinib	Compound	99.97	Heart Diseases (Clinical) [3]; Asthma (Clinical) [71]; Insulin Resistance (Clinical) [72].
12	Lorazepam	Compound	99.61	Anxiety Disorders (FDA-approved).
13	Parecoxib	Compound	99.56	Pain (FDA-approved); Inflammation (FDA-approved).
14	KUC103904N	Compound	99.51	Not detected.
15	Cefalexin	Compound	99.51	Surgical Wound Infection (FDA-approved); Urinary Tract Infections (FDA-approved); Respiratory Tract Infections (FDA-approved); Skin Diseases, Infectious (FDA-approved); Otitis Media (FDA-approved).
16	SNS-314	Compound	99.37	Pulmonary Fibrosis (Clinical) [73]; Arthritis, Rheumatoid (Clinical) [74]; Alzheimer’s Disease (Preclinical) [13]; Psoriasis (Preclinical) [75].
17	Pioglitazone	Compound	99.28	Diabetes Mellitus; Insulin Resistance (FDA-approved).
18	GSK-1904529A	Compound	99.12	None found.
19	BRD-A97035593	Compound	99.09	None found.
20	Tandutinib	Compound	99.07	Parkinson’s Disease (Preclinical) [2].
21	STAT4	Gene (kd)	99.34	Autoimmune Diseases (Clinical) [76]; Inflammation (Clinical); (Preclinical) [77]; Hypersensitivity (Preclinical) [77].
22	YTHDF2	Gene (kd)	99.29	Alzheimer’s Disease (Preclinical) [78].
23	ZNF92	Gene (kd)	99.21	Alzheimer’s Disease (Preclinical) [79]; Parkinson’s Disease (Preclinical) [79].
24	C2	Gene (oe)	99.12	Systemic Lupus Erythematosus (Clinical) [80]; Sjögren’s Syndrome (Clinical) [80].
25	CLPB	Gene (oe)	99.07	Neutropenia (Clinical) [81]; Parkinson’s Disease (Preclinical) [82].
26	KIF5C	Gene (kd)	99.03	Neurodegenerative Diseases (Preclinical) [83].
27	TKT	Gene (oe)	98.98	Diabetes Mellitus (Preclinical) [84]; Alzheimer’s Disease (Preclinical) [85].
28	ACAT1	Gene (kd)	98.82	Hypertension (Preclinical) [86]; Dementia (Preclinical) [87].
29	SLC3A2	Gene (oe)	98.74	Wound Healing (Preclinical) [88].
30	YTHDF1	Gene (kd)	98.74	Diabetes Mellitus (Clinical) [89]; Alzheimer’s Disease (Preclinical) [90].

Non-cancer indications not identified in the literature or publicly available sources, as of 28 April 2025, are denoted as ‘None found’. Validity levels are described in the Methods section.

The frequency analysis of the non-cancer indications of strong CMap positive-scoring connections indicated that imatinib exhibits a broad systemic pharmacology, consistent with its known off-target actions affecting immune cells, vasculature, and metabolism. This analysis highlighted Alzheimer’s disease (seven times), Parkinson’s disease (six times), diabetes mellitus (four times), hypertension (four times), inflammation (three times), schizophrenia (two times), heart diseases (two times), and insulin resistance (two times). Other non-cancer indications that appeared only once are listed in Table 2. These findings are consistent with imatinib’s multi-kinase inhibition profile, particularly against PDGFR, c-KIT, and ABL kinases, and suggest broad repositioning opportunities across CNS, cardiovascular, metabolic, and autoimmune conditions.

#### 2.3.3. Non-Cancer Indications of Nilotinib

Nilotinib showed a tight clustering of connections, specifically with aurora kinase inhibitors, heat shock protein inhibitors, and other BCR-ABL or tyrosine kinase-related perturbagens. Its compound connectivity profile is extremely specific, with perfect (score = 100) or near-perfect similarity to related kinase inhibitors. Gene-level connectivity includes genes directly involved in cell cycle regulation and kinase signaling, reinforcing its focused biological activity and minimal off-target effects.

The frequency analysis of non-cancer indications (using MeSH terms) indicated that nilotinib’s top CMap positive-scoring perturbagen connections are strongly enriched for neurological, autoimmune, fibrotic, and inflammatory diseases (Table 3). This analysis highlighted Alzheimer’s disease (six times), autoimmune diseases (three times), diabetes mellitus (three times), Parkinson’s disease (three times), and pulmonary fibrosis (three times). Other notable indications included amyotrophic lateral sclerosis, rheumatoid arthritis, COVID-19, ulcerative colitis, inflammation, leukoencephalopathy, nervous system diseases, neurodegenerative diseases, and psoriasis, reflecting a diverse enrichment across neurodegenerative, autoimmune, inflammatory, and infectious disease categories. These findings regarding nilotinib’s polypharmacology are consistent with previous evidence, which highlighted neurodegeneration and immunomodulation as key non-cancer repositioning opportunities [91]. However, this study suggests more repurposing opportunities and underscores the importance of multi-pathway inhibitors in identifying therapeutic strategies for complex diseases.

**Table 3:** Top CMap positive-scoring perturbagen connections to nilotinib and their known non-cancer indications.

#	Perturbagen	Type	Score	Non-Cancer Indications
1	Aurora Kinase inhibitor Grp1	Class	99.90	Alzheimer’s Disease (Preclinical) [13]; Liver Cirrhosis, Experimental (Preclinical) [92]; Pulmonary Fibrosis (Preclinical) [14].
2	JAK inhibitor	Class	99.28	Alopecia Areata (FDA-approved); Colitis, Ulcerative (FDA-approved); Crohn’s Disease (FDA-approved); Vitiligo (FDA-approved); Arthritis, Rheumatoid (Clinical) [93]; Autoimmune Diseases (Clinical) [94]; Arthritis (Clinical) [95]; Psoriasis (Clinical) [95]; Dermatitis, Atopic (Clinical) [96]; Diabetes Mellitus (Clinical) [97].
3	Vesicular transport	Class	99.28	None found.
4	EGFR inhibitor	Class	99.09	Psoriasis (Preclinical) [98]; Pulmonary Fibrosis (Preclinical) [99]; Atherosclerosis (Preclinical) [100]; Neovascularization, Pathologic (Preclinical) [101].
5	BRAF RAF1 inhibitor	Class	98.91	Pulmonary Fibrosis (Preclinical) [28]; Arthritis, Rheumatoid (Preclinical) [27].
6	EIF proteins	Class	98.73	Leukoencephalopathy (Preclinical) [102]; Diabetes Mellitus (In silico) [103].
7	NFKB activation	Class	98.57	Inflammation (Clinical) [104]; Neurodegenerative Diseases (Preclinical) [105].
8	BCL2 and related protein inhibitor	Class	98.33	Alzheimer’s Disease (Preclinical) [106]; Lupus Erythematosus, Cutaneous (Preclinical) [107];
9	GSK3 inhibitor	Class	98.23	Psychiatric Disorders (Clinical) [108]; Alzheimer’s Disease (Preclinical) [109]; Diabetes Mellitus (Clinical) [110]; Inflammation (Preclinical) [111].
10	RAR agonist Grp2	Class	98.21	Acne Vulgaris (Clinical) [112].
11	Nilotinib	Compound	99.99	Parkinson’s Disease (Clinical) [7]; Alzheimer’s Disease (Clinical) [113]; Amyotrophic Lateral Sclerosis (Preclinical) [114].
12	AT-9283	Compound	99.79	Hypertension, Pulmonary (Preclinical) [115]; COVID-19 (In silico) [116].
13	Alisertib	Compound	99.68	None found.
14	ZM-447439	Compound	99.61	None found.
15	Avrainvillamide-analog-2	Compound	99.58	Antibacterial Activity (Preclinical) [117].
16	Tozasertib	Compound	99.40	Optic Nerve Injuries (Preclinical) [118].
17	Crizotinib	Compound	99.38	None found.
18	Erastin	Compound	99.30	None found.
19	KI-8751	Compound	99.19	Cystitis (Preclinical) [119].
20	MK-5108	Compound	99.12	Kidney Fibrosis (Preclinical) [120].
21	HSPA5	Gene (kd)	99.84	Fatty Liver, Nonalcoholic (Clinical) [121]; Alzheimer’s Disease (Preclinical) [122]; Parkinson’s Disease (Preclinical) [123]; Myositis (Preclinical) [124]; COVID-19 (Preclinical) [125].
22	COPA	Gene (kd)	99.84	Autoimmune Diseases (Clinical) [126].
23	GMDS	Gene (oe)	99.81	Glaucoma, Open-Angle (Preclinical) [127].
24	SFPQ	Gene (oe)	99.77	HIV Infection (Clinical) [128]; Nervous System Diseases (Preclinical) [129]; Congenital Structural Myopathies (Preclinical) [130]; Amyotrophic Lateral Sclerosis (Preclinical) [131].
25	HSP90B1	Gene (kd)	99.73	Tuberculosis (Preclinical) [132]; Polycystic Ovary Syndrome (Preclinical) [133].
26	HRSP12	Gene (oe)	99.68	Osteoarthritis (Preclinical) [134]; Diabetic Nephropathies (In silico) [135].
27	EIF2B2	Gene (kd)	99.66	Leukoencephalopathy (Preclinical) [136]; Nervous System Diseases (Preclinical) [137].
28	NIT1	Gene (kd)	99.63	None found.
29	NFE2L2	Gene (oe)	99.63	Parkinson’s Disease (Preclinical) [138]; Heart Failure (Preclinical) [139].
30	LYN	Gene (oe)	99.63	Autoimmune Diseases (Preclinical) [140]; Neurodegenerative Diseases (Preclinical) [140]; Alzheimer’s Disease (Preclinical) [141].

If no non-cancer indications were identified in the published literature or open-source databases, as of 28 April 2025, the designation “None found” was assigned. Validity levels are described in the Methods section.

### 2.4. Supporting Evidence

Supporting evidence from network biology analyses and/or the biomedical literature is a valuable layer of validation for computational hypotheses. The reliability of such an approach depends on the source quality, context, and convergence with other evidence. Thus, manual curation and critical evaluations are essential.

#### 2.4.1. BCR-ABL PPI Network and Annotated Enriched Pathways

**Figure 5:**
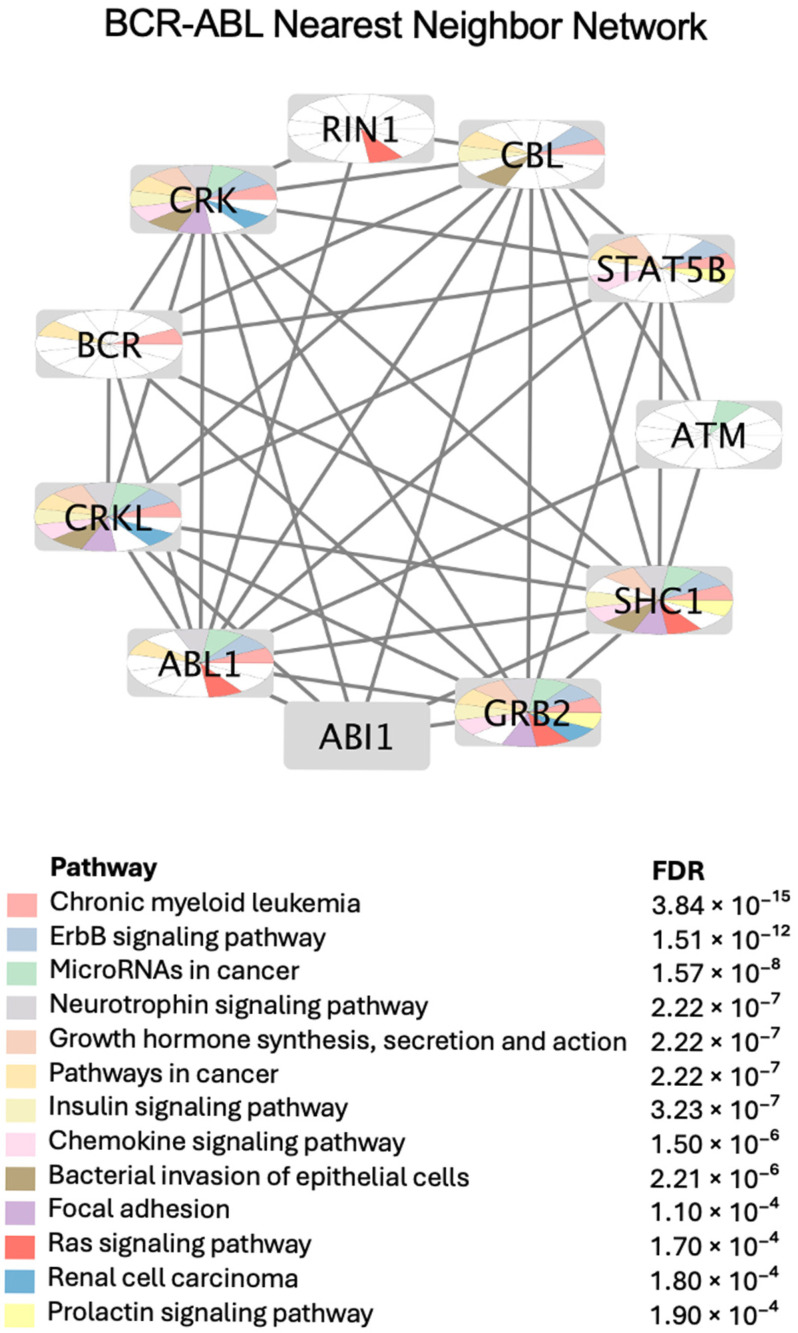
BCR-ABL protein–protein interaction (PPI) network and pathway enrichment. The network was generated using BCR-ABL as the input. Nodes represent proteins directly interacting with BCR-ABL, and edges indicate known or predicted protein–protein associations. Node segments are color-coded based on the top 10 enriched KEGG pathways identified through over-representation analysis in Cytoscape. White colors indicate that the corresponding pathways are not enriched in that node. A totally grey node indicate that this node was not enriched in any of the top pathways shown in this figure.

BCR-ABL is a fusion oncogene that is characteristic of CML, whereas BCR and ABL1 are separate genes. Most pathway databases treat BCR-ABL as a functional complex. As shown in Figure 5, BCR-ABL inhibitors not only modulate leukemic signaling but also key hubs such as STAT5, GRB2, and CBL. Enrichment in pathways like ErbB, insulin signaling, and focal adhesion aligns with CMap predictions, suggesting potential for repurposing these inhibitors in neurodegenerative diseases, fibrosis, and metabolic disorders. These findings underscore the broader therapeutic relevance of BCR-ABL beyond CML.

#### 2.4.2. Literature-Based Validation

Most BCR-ABL inhibitors, including imatinib, nilotinib, dasatinib, bosutinib, tozasertib, and AT-9283, are FDA-approved for the treatment of hematological malignancies such as leukemia—myelogenous, chronic, BCR-ABL-positive (CML); acute lymphocytic leukemia (ALL); mastocytosis; gastrointestinal stromal tumors; hypereosinophilic syndrome; and other related conditions. In addition to their established oncological uses, these inhibitors have also been associated with non-cancer indications at various stages of validation, including clinical, preclinical, and hypothesis-driven studies as reported in Table 4. These emerging applications span across areas such as neurodegeneration, inflammation, fibrosis, and metabolic dysfunction. Thus, these literature-based findings confirm that BCR-ABL inhibitors exhibit strong non-cancer repositioning potential toward neurodegenerative, autoimmune, fibrotic, metabolic, and infectious diseases. Therefore, this serves as additional proof that some CMap-predicted indications align closely with known clinical and preclinical observations, supporting the robustness of connectivity mapping for drug repurposing hypotheses. Other unreported indications predicted from the CMap could serve as robust novel hypotheses.

**Table 4:** Cancer and non-cancer indications for BCR-ABL inhibitors and drug targets.

#	Perturbagen	Cancer Indications and Validity	Non-Cancer Effects and Validity
1	AT-9283	Leukemia, Lymphoid (Clinical) [142]; Multiple Myeloma (Clinical) [143]; Neoplasms (Clinical) [144]; Lymphoma, B-Cell (Preclinical) [145].	Myeloproliferative Disorders (Preclinical) [146].
2	Bosutinib	Leukemia, Myelogenous, Chronic, BCR-ABL Positive (FDA-approved).	Lewy Body Disease (Clinical) [147].
3	Dasatinib	Leukemia, Myelogenous, Chronic, BCR-ABL Positive (FDA-approved).	Alzheimer’s Disease (Clinical) [148]; COVID-19 (Clinical) [149]; Hepatitis B, Chronic (Clinical) [150]; Pulmonary Fibrosis (Clinical) [151]; Obesity (Preclinical) [152].
4	HY-1107	Liver Neoplasms (Preclinical)	None found.
5	Imatinib	Leukemia, Lymphoid (FDA-approved); Mastocytosis (FDA-approved); Leukemia, Myelogenous, Chronic, BCR-ABL Positive (FDA-approved); Dermatofibrosarcoma Protuberans (FDA-approved); Hypereosinophilic Syndrome (FDA-approved); Leukemia, Eosinophilic, Chronic (FDA-approved); Gastrointestinal Stromal Tumors (FDA-approved); Myeloproliferative Disorders (FDA-approved).	Anemia, Sickle Cell (Clinical) [153]; Diabetes Mellitus, Type 1 (Clinical) [154]; Pulmonary Fibrosis (Clinical) [155]; Stroke (Clinical) [156]; Liver Cirrhosis (Clinical) [157].
6	Nilotinib	Leukemia, Myelogenous, Chronic, BCR-ABL Positive (FDA-approved).	Alzheimer’s Disease (Clinical) [158]; Parkinson’s Disease (Clinical) [7].
7	Tozasertib	Glioma (Clinical) [159]; Melanoma (Preclinical) [160].	Hypersensitivity (Preclinical) [161]; Neuralgia (Preclinical) [162].
8	BCR-ABL	Leukemia, Myelogenous, Chronic, BCR-ABL Positive (Clinical) [163].	None found.
9	BCR	None found.	Autoimmune Diseases (Clinical) [164].
10	ABL	None found.	Developmental Disabilities (Preclinical) [165]; Parkinson’s Disease (Preclinical) [166].

If no indications were identified in the published literature or open-source databases, as of 28 April 2025, the designation “None found” was assigned. Validity levels are described in the Methods section.

The strong positive CMap associations of imatinib and nilotinib with non-oncological indications suggest high-confidence repurposing opportunities warranting clinical investigation. Imatinib’s robust connectivity with PDGFR and c-KIT indicates its potential efficacy in treating pulmonary fibrosis. PDGFR-α/β signaling is known to drive fibroblast proliferation, migration, and extracellular matrix production, processes which are central to fibrotic progression; thus, its inhibition can attenuate these fibrotic responses [167]. Similarly, c-KIT facilitates the recruitment of bone marrow-derived progenitor cells that differentiate into myofibroblasts, contributing to fibrosis; inhibiting c-KIT may reduce this pathogenic cell population [168].

Nilotinib’s strong positive connectivity with HSP90, HSPA5, and LYN suggests repurposing potential for Alzheimer’s disease. HSP90, HSPA5, and LYN are implicated in key Alzheimer’s disease pathologies, including neuroinflammation and disrupted protein homeostasis. Evidence indicates that nilotinib may exert neuroprotective effects by promoting the autophagic clearance of misfolded proteins, reducing endoplasmic reticulum (ER) stress, and modulating the unfolded protein response (UPR) pathways [169,170]. Nilotinib’s inhibition of HSP90 could reduce tau phosphorylation and amyloid-β accumulation, further supporting its therapeutic potential in Alzheimer’s disease. It is known that HSP90 activation drives the production of pathological tau aggregates [171]. Furthermore, it has been reported that the administration of Hsp90 inhibitors could prevent Aβ-induced neurotoxicity by increasing the levels of HSP70 and Hsp90 [172].

### 2.5. Mechanistic Insight

**Figure 6:**
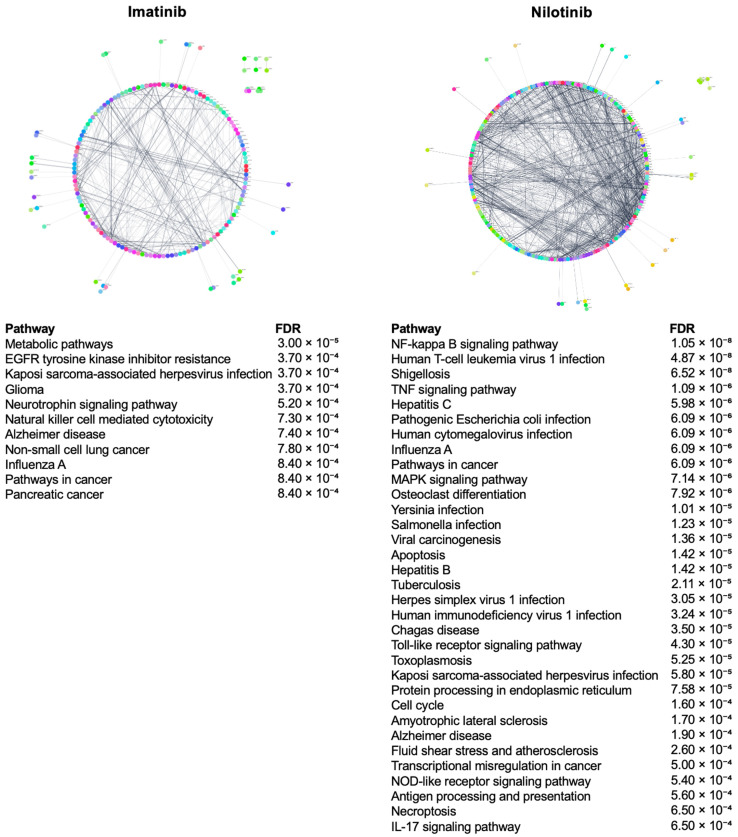
Comparative PPI networks and enriched pathways of imatinib’s and nilotinib’s positive CMAP connections, revealing divergent polypharmacological profiles. Networks were created using all positive gene connections with CMap scores ≥ 90.00.

The analysis of strong positive gene connections with CMap scores ≥ 90.00 for both imatinib and nilotinib (Supplementary Tables S2 and S3), using protein–protein interaction networks and pathway enrichment analysis, revealed distinct biological signatures for each compound (Figure 6). A complete list of enriched pathways is provided in Supplementary Tables S4 and S5.

Imatinib’s associated network is comparatively sparse and modular, with a lower connectivity among gene products. This pattern suggests a broader and less convergent transcriptional response, potentially involving multiple biological domains without a single dominant signaling axis. The top KEGG-enriched pathways linked to imatinib include EGFR signaling, glioma, neurotrophin signaling, and various metabolic processes.

In contrast, the PPI network associated with nilotinib is highly interconnected, indicating a focused and coherent biological response. Its strong enrichment in KEGG immune-related pathways, including TNF, NFκB, Toll-like receptor, IL17, and NOD-like receptor signaling, highlights alignment with core immunoregulatory mechanisms.

## 3. Discussion

This study presents a comprehensive informatics workflow for investigating the network pharmacology of BCR-ABL inhibitors, leveraging cheminformatics, transcriptomics, and literature mining processes to uncover their polypharmacological potential beyond oncology. The results highlight structural and biological diversity among these inhibitors, their broad transcriptional effects, and their potential repurposing for non-cancer indications.

A cheminformatics analysis revealed significant chemical diversity among the seven BCR-ABL inhibitors, with distinct structural clusters identified by Tanimoto fingerprint and Euclidean distance metrics. Nilotinib and imatinib showed the highest similarity, while HY-11007 and AT-9283 were structurally distinct, as confirmed by PCA. Integrating fingerprint- and descriptor-based metrics uncovered orthogonal dimensions of similarity, supporting the hypothesis that structural diversity underlies the polypharmacologic and off-target profiles of these inhibitors. Compounds with high DLS_cons values clustered within favorable drug-like chemical spaces, whereas structurally distant compounds, though potentially potent, may require formulation or ADMET optimization. These findings highlight the value of integrated cheminformatics approaches in modern drug design, network pharmacology, and precision medicine.

Biologically, the BCR-ABL inhibitors exhibited varying selectivity and polypharmacology. Nilotinib was the most selective, while dasatinib and bosutinib showed broader kinase inhibition, including SRC family targets. Despite structural similarity, HY-11007 displayed minimal transcriptional activity, indicating limited cellular engagement. CMap analysis corroborated these patterns, with dasatinib and nilotinib inducing strong transcriptional responses, whereas HY-11007 showed weak activity. These findings highlight the importance of integrative approaches that consider both structural and functional profiles when repurposing kinase inhibitors.

The CMap analysis revealed extensive polypharmacology among BCR-ABL inhibitors, with strong connections to unrelated drug classes (e.g., EGFR inhibitors and VEGFR inhibitors) and genes involved in diverse pathways (e.g., PI3K/AKT and RAS signaling). These findings align with their known multi-kinase activities and suggest potential repurposing opportunities for non-cancer diseases. Key non-cancer indications for BCR-ABL inhibitors include neurodegenerative diseases, with Alzheimer’s and Parkinson’s diseases frequently being associated, particularly for nilotinib and dasatinib, which have shown preclinical and clinical neuroprotective effects. Fibrotic disorders such as pulmonary and liver fibrosis also emerged, likely reflecting the inhibition of PDGFR and other pro-fibrotic kinases. Additionally, links to autoimmune and inflammatory diseases, including rheumatoid arthritis and psoriasis, were supported by connections to JAK inhibitors and immune-modulatory genes like STAT4. Selective JAK inhibitors such as baricitinib have been primarily developed and approved for rheumatoid arthritis, COVID-19 (emergency use) [173], and atopic dermatitis. Metabolic disorders, notably diabetes mellitus and insulin resistance, were also associated, possibly through the modulation of insulin signaling pathways.

Notably, both imatinib and nilotinib are BCR-ABL inhibitors; their non-cancer profiles diverged markedly. Imatinib exhibited broad systemic effects, with strong associations to cardiovascular (hypertension), metabolic (diabetes), and neuropsychiatric (schizophrenia and depression) conditions, consistent with its multi-kinase inhibition profile, targeting PDGFR and c-KIT. In contrast, nilotinib showed more focused activity in neurodegeneration (Alzheimer’s and Parkinson’s) and autoimmune diseases, reflecting its higher selectivity and unique off-target interactions, such as HSP90 inhibition. These differences underscore how structural features and target selectivity shape repurposing potential, with imatinib offering versatility but greater off-target risks, and nilotinib presenting a more favorable profile for CNS-focused applications.

These predictions serve as repurposing hypotheses for BCR-ABL inhibitors. Predictions were supported by evidence from the literature, as reported in Table 1, Table 2 and Table 3, where many CMap-identified indications matched known preclinical or clinical findings. For example, imatinib’s anti-fibrotic effects in pulmonary fibrosis and nilotinib’s neuroprotective role in Parkinson’s disease are well-documented (references are cited in Table 4). This convergence of computational and experimental data strengthens the validity of the hypotheses generated. Furthermore, the functional protein–protein interaction (PPI) network analysis (Figure 5) revealed that BCR-ABL inhibitors modulate key hubs beyond oncogenic signaling (e.g., STAT5 and GRB2). Enriched pathways included ErbB signaling, insulin signaling, and focal adhesion, which are implicated in neurodegeneration, fibrosis, and metabolic disorders. This system-level perspective further reinforces the CMap predictions and provides mechanistic insights into how these inhibitors might exert therapeutic effects in non-cancer contexts.

A deeper analysis of CMap gene connections for imatinib and nilotinib, using protein–protein interaction networks and pathway enrichment, highlighted distinct mechanistic profiles. Imatinib’s enrichment in EGFR signaling reflects its broader polypharmacological activity and less-selective targeting of BCR ABL. Given the emerging role of EGFR as a central mediator of profibrotic programs across organs, often acting upstream of TGF beta1-driven fibroblast activation [174], imatinib may hold potential as an antifibrotic agent warranting further investigation. In contrast, nilotinib showed strong enrichment in immune-related pathways such as TNF, NFκB, Toll like receptor, IL17, and NOD-like receptor signaling. These pathways are closely linked to neuroinflammatory mechanisms in central nervous system disorders, including Alzheimer’s and Parkinson’s disease [174], suggesting that nilotinib may modulate microglial activation and reduce disease-associated proteinopathies, supporting its repurposing potential in Alzheimer’s and Parkinson’s diseases.

### 3.1. Translational and Safety Implications

This hypothesis-generating study investigates new therapeutic opportunities for BCR ABL inhibitors beyond oncology. By integrating chemical, transcriptomic, and pathway level data, we identified mechanistic overlaps with key processes in fibrosis, neurodegeneration, and immune-mediated diseases. These findings provide a framework for repurposing approved drugs for conditions with limited treatment options. While not immediately applicable to clinical practice, this work lays the groundwork for future trials by offering testable hypotheses and mechanistic insights to support preclinical validation and targeted therapy design. Our approach may also reduce uncertainty, cost, and timelines in early-stage drug development.

We also acknowledge that kinase inhibitors often present complex safety profiles due to their polypharmacological nature and off-target effects. For example, nilotinib has been associated with an increased risk of cardiovascular events, including arterial occlusion, myocardial ischemia, and stroke, while imatinib is frequently linked to fluid retention, leading to periorbital and generalized edema [175,176]. Although a full in silico toxicogenomic assessment was beyond the scope of this study, we recognize the value of incorporating predictive safety tools into future workflows. This will allow for a more comprehensive evaluation of the risk–benefit profiles of repurposing candidates and further enhance their translational readiness.

### 3.2. Limitations

Despite the strengths of our integrative workflow, several limitations should be noted. The CMap platform uses immortalized cancer cell lines, which may not reflect the tissue-specific context of diseases like fibrosis or neurodegeneration. Its focus on transcriptomic data excludes proteomic and phenotypic layers that are essential for full mechanistic insight. Variability in cell lines, dosing, and timing may also affect reproducibility, and reliance on inferred expression from a limited set of landmark genes could introduce errors in pathway analysis.

### 3.3. Future Perspectives

Realizing the translational potential of BCR ABL inhibitors beyond oncology will require coordinated, multidisciplinary efforts. Future research should focus on validating the most promising non-oncologic indications through preclinical studies and targeted clinical trials. High-throughput multi-omics approaches, including transcriptomics, proteomics, and metabolomics, will be essential for mapping the systemic effects of these compounds across disease models, particularly in fibrotic, neurodegenerative, and immune-mediated conditions. Integrating machine learning with systems pharmacology may accelerate the discovery of repurposing opportunities and improve the prediction of adverse events. Additionally, leveraging pharmacogenomic and real-world data will support patient stratification and guide the development of personalized therapeutic strategies.

## 4. Methods

### 4.1. Chemical Biology Informatics Workflow

We applied an informatics workflow, based on the methods developed by Hajjo et al. [9,10,11,12]. The bioinformatics workflow method was used to study the network biology of BCR-ABL inhibitors. This workflow involved the following three steps: (1) identifying gene signatures representative of the chemical compound’s biological action; (2) identifying genetic perturbations that result in transcriptomics profiles resembling those of BCR-ABL inhibitors; and (3) a pathway enrichment and literature mining process to elucidate the biological activities of similar gene and compound connections, respectively. Furthermore, cheminformatics methods were employed to assess and compare chemical similarities/dissimilarities between BCR-ABL inhibitors.

In this study, Connectivity Map (CMap) was used to identify transcriptomic signatures and prioritize repurposing hypotheses for BCR ABL inhibitors through the following steps:

1. Querying CMap: Gene expression signatures were retrieved using the search terms “mechanism = BCR ABL inhibitors”, “compound = imatinib”, and “compound = nilotinib”.
2. Generating query signatures: The transcriptomic profiles of compounds identified from the initial search were used as input query signatures.
3. Identifying similar perturbations: These signatures were used to search CMap for compounds and genetic perturbations exhibiting similar expression profiles.
4. Prioritization of hits: Repurposing hypotheses were prioritized based on the top 10 gene and compound connections, all enriched pharmacological classes, and supporting preclinical or clinical evidence.
5. Filtering by connectivity scores: The results were filtered using CMap connectivity scores, as previously described [8], to retain only relevant and strong associations.
6. Pathway enrichment: High-confidence gene connections (CMap scores ≥ 90.00) were used for pathway enrichment analysis to gain mechanistic insights.

### 4.2. Bioinformatics Methods

#### 4.2.1. The Connectivity Map (CMap)

The CMap computational tool and a chemogenomics database [8] constitute a cornerstone of the chemocentric informatics workflow developed by Hajjo et al. [9,10,11,12]. This tool was used to formulate testable hypotheses by reading compounds, genes, and compound classes that share a strong similarity with BCR-ABL inhibitors on the transcriptomics level. The CMap database lists 1.3 million profiles of transcriptional responses of human cells to chemical and genetic perturbations. Currently, there are 27,927 perturbagens (19,911 small molecules and 7494 genetic perturbagens) generating 476,251 expression signatures in nine human cell lines—PC3, VCAP, A375, A459, HA1E, HCC515, HT29, MCF7, and HEPG2. This database uses the L1000 platform version 1.0 [8], a high-throughput gene expression assay that measures the mRNA transcript abundance of 978 “landmark” genes in human cells.

#### 4.2.2. Protein–Protein Interactions

A systematic search for BCR-ABL nearest neighbor (NN) genes/proteins was conducted in Cytoscape version 3.10.3, using the STRING App version 2.1.0 [177]. All retrieved protein–protein interactions (PPIs), including both physical and functional associations, were collected using a medium confidence threshold of 0.4. In STRING, a confidence score of 0.4 reflects a moderate level of predicted interaction reliability, balancing sensitivity and specificity by including experimentally supported and computationally inferred interactions. The resulting data were then used to generate PPI networks using Cytoscape’s network visualization and analysis tools, followed by enrichment analysis-leveraging modules from Gene Ontology (Biological Process, Molecular Function, and Cellular Component), KEGG, Reactome, WikiPathways, protein domain annotations, DISEASES, COMPARTMENTS, and TISSUES.

Furthermore, PPI networks for high-confidence gene connections (CMap scores ≥ 90.00) of imatinib and nilotinib, generated using Cytoscape’s STRING App version 2.1.0, were used for pathway enrichment analysis to gain mechanistic insights.

#### 4.2.3. Enrichment Analysis

Pathway enrichment analyses for PPI networks were performed using the STRING functional enrichment tool in Cytoscape [177] (version 3.10.3). The significance of each enrichment result was assessed by calculating hypergeometric *p*-values. Next, all ontology terms were ranked according to their calculated false discovery rates (FDRs). Pathways with FDRs below a threshold of 0.05 were considered statistically significant and were selected for further biological investigation.

### 4.3. Cheminformatics Methods

#### 4.3.1. Molecular Descriptors

Two-dimensional (2D) molecular descriptors from alvaDesc version 1.0.18 [178] and circular fingerprints (Morgan fingerprints) were generated for BCR-ABL inhibitors. The 2D chemical structures of seven inhibitors were first retrieved from PubChem and exported in standardized Structure Data File (SDF) format. Molecular standardization was performed using RDKit version 2024.03.1 [179], in the Python environment version 3.12.2, to ensure consistency in structural representation. This process included the removal of salts, charge neutralization, tautomer canonicalization, aromatic ring perception (aromatization), and the addition of explicit hydrogen atoms.

#### 4.3.2. Similarity and Distance Matrix Calculation

Pairwise molecular similarities were computed using the following two approaches: (1) Tanimoto coefficients with circular fingerprints (Morgan fingerprints) generated using RDKit, and (2) Euclidean distances with 2D alvaDesc molecular descriptors. The Tanimoto distance was calculated as 1–Tanimoto Similarity, yielding a symmetric distance matrix assembled using pandas version 2.2.2. The distance matrix was visualized using a heatmap generated with Seaborn version 0.13.2 (sns.heatmap) and Matplotlib version 3.8.4. All generated plots were saved in TIFF format.

### 4.4. Standardization of Condition Terms Using MeSH Vocabulary

Condition terms in all tables were standardized using Medical Subject Headings (MeSH)-controlled vocabulary to ensure terminological consistency. All condition variants (e.g., user-defined, literature-derived, or dataset-specific) were converted to standard descriptors (i.e., MeSH IDs) via automated mapping using the MeSH Resource Description Framework (MeSH RDF) [180].

### 4.5. Therapeutic Evidence

To systematically assess the confidence and novelty of repurposing candidates, we categorized all perturbagens (including small molecules and genetic perturbations) into four hierarchically ordered evidence levels based on translational validation, as follows:

1. FDA-approved: Interventions that are already approved for non-cancer indications. These reflect previously validated clinical uses and are not novel findings of this study but were included for benchmarking and validation purposes.
2. Clinical: Candidates with reported efficacy in human studies or clinical trials but without formal regulatory approval. These are supported by clinical evidence yet still represent off-label or investigational use.
3. Preclinical: Agents with supportive data from animal models or in vitro experiments but lacking human clinical validation.
4. In silico/Hypothesis: Computational predictions generated in this study that have not yet been tested experimentally.

This tiered framework enabled a structured prioritization of repurposing hypotheses, balancing novelty with confidence. It was consistently applied across all reported associations and is visually encoded in the tables and figures to clearly distinguish between novel predictions and previously validated indications, thereby enhancing interpretability and translational relevance.

## 5. Conclusions

This study presents an integrative informatics framework that effectively maps the polypharmacology of BCR-ABL inhibitors, highlighting their potential for repurposing in neurodegenerative, fibrotic, autoimmune, and metabolic diseases. The convergence of cheminformatics, transcriptomics, and evidence from the literature demonstrates the power of computational methods in drug repositioning. Notably, imatinib and nilotinib emerged as promising candidates for diverse non-cancer applications, with their distinct profiles offering complementary therapeutic opportunities. In this approach, CMap enabled the identification of biological similarities with imatinib and nilotinib based on transcriptomics effects. In the last step, literature mining identified the biological activities of compound hits, while enrichment analysis identified enriched biological pathways for the identified gene hits, which served as robust supporting evidence for the polypharmacology hypotheses derived from CMap. These findings support the rationale for targeted experimental validation and clinical investigation to translate computational hypotheses into real-world therapies. Moreover, this integrative approach provides a broadly applicable model for studying the polypharmacological effects of other classes of inhibitors.

## Figures and Tables

**Table 1:** Top CMap positive-scoring perturbagen connections to BCR-ABL inhibitors and their known non-cancer indications.

#	Perturbagen	Type	Score	Non-Cancer Indications and Validity
1	Aurora kinase inhibitor	Class	99.46	Alzheimer’s Disease (Preclinical) [13]; Pulmonary Fibrosis (Preclinical) [14]; Inflammatory Bowel Diseases (Preclinical but weak) [15]; Arthritis, Psoriasis (Preclinical) [16]; Arthritis, Rheumatoid (In silico) [17].
2	PDGFR\KIT inhibitor	Class	99.34	Alzheimer’s Disease (Preclinical) [18]; Atherosclerosis (Preclinical) [19]; Gastrointestinal Diseases (Preclinical) [18]; Liver Cirrhosis, Experimental (Preclinical) [18] Pulmonary Fibrosis (Preclinical) [20]; Wound Healing (Preclinical) [18].
3	EGFR inhibitor	Class	99.02	Pulmonary Fibrosis (Preclinical) [21]; Psoriasis (Preclinical) [22].
4	VEGFR inhibitor	Class	98.73	Eye Diseases (Clinical/Approved) [23]; Arthritis, Rheumatoid (Preclinical) [24]; Hypertension, Portal [25]; Psoriasis (Preclinical) [26].
5	RAF inhibitor	Class	98.67	Arthritis, Rheumatoid (Preclinical) [27]; Lung Injury and Pulmonary Fibrosis (Preclinical) [28].
6	Afatinib	Compound	99.44	Skin Diseases (Preclinical) [29].
7	HG-6-64-01	Compound	99.40	Heart failure (Preclinical) [30].
8	Carnertinib	Compound	99.26	Liver Cirrhosis, Experimental (Preclinical) [31].
9	Tetrindole	Compound	99.19	Depression (Preclinical/Early clinical) [32].
10	WZ-4-145	Compound	99.19	Unknown.
11	SU-11652	Compound	99.12	Unknown. Multi-targeted TKI.
12	Mibefradil	Compound	98.98	Pain, Neuropathic (Preclinical) [33].
13	NVP-TAE684	Compound	99.98	Neuroprotection; Alzheimer Disease (Preclinical) [34].
14	Cediranib	Compound	98.94	Liver Cirrhosis, Experimental (Preclinical) [25].
15	AZD-7762	Compound	98.93	Osteoporosis (Preclinical) [35].
16	TERF1	Gene (kd)	98.77	Aging (Preclinical) [36]; Alzheimer’s Disease (Preclinical) [37]; Infertility, Male (Preclinical) [38].
17	C9ORF96	Gene (kd)	98.62	Unknown.
18	UGCG	Gene (oe)	98.10	Gaucher Disease (Clinical) [39]; Keloid (Preclinical) [40]; Myocardial Fibrosis (Preclinical) [41]; Vascular Malformations (Preclinical) [42]; Parkinson’s Disease (Preclinical) [43], Depressive Disorder (Preclinical) [43].
19	AKT3	Gene (kd)	97.47	Brain Malformations (Clinical) [44]; Autoimmunity (Preclinical) [45]; Cognitive Dysfunction (Preclinical) [46]; Hemimegaloencephaly (Preclinical) [47]; Wound Healing (Preclinical) [48].
20	ZNF449	Gene (kd)	97.60	Chondrogenesis/Cartilage (Preclinical) [49].
21	KRAS	Gene (kd)	97.50	Kidney Fibrosis (Preclinical) [50].
22	FABP4	Gene (oe)	97.32	Diabetes Mellitus (Clinical) [51]; Atherosclerosis (Preclinical) [52]; Kidney Stones (Preclinical) [53]; Obesity (Preclinical) [54].
23	PTK2	Gene (kd)	97.22	Alzheimer’s Disease (Preclinical) [55].
24	AK3	Gene (kd)	97.14	Alzheimer’s Disease (In silico) [56].
25	MRP1L18	Gene (oe)	97.12	Asthma (Preclinical) [57].

The table lists the top-scoring compounds, drug classes, and gene perturbations based on transcriptomic similarity scores. Entries include their known cancer and non-cancer indications, highlighting potential polypharmacology and repurposing opportunities. Literature-based evidence is provided for non-cancer indications. Non-cancer indications not identified in the literature or publicly available sources, as of 28 April 2025, are denoted as ‘None found’. Validity levels are described in the Methods section.

## Data Availability

The raw cheminformatics descriptors, transcriptomics profiles from CMap analysis, and processed network pharmacology datasets generated in this study are available on GitHub at [https://github.com/rhajjo/BCR-ABL] (accessed on 15 June 2025). These data should enable the full reproducibility of the reported findings.

## Supplementary Materials

The following supporting information can be downloaded at https://www.mdpi.com/article/10.3390/ph18070936/s1. Supplementary Table S1: List of calculated 2D molecular descriptors generated using alvaDesc. Supplementary Table S2: CMap-derived positive gene expression connections for imatinib (CMap scores ≥ 90.00). Supplementary Table S3: CMap-derived positive gene expression connections for nilotinib (CMap scores ≥ 90.00). Supplementary Table S4: Enriched pathway maps based on the PPI network of imatinib’s CMap-positive gene connections (CMap scores ≥ 90.00). Supplementary Table S5: Enriched pathway maps based on the PPI network of nilotinib’s CMap-positive gene connections (CMap scores ≥ 90.00).

## Abbreviations

ABL	Abelson Murine Leukemia Viral Oncogene
ALK	Anaplastic Lymphoma Kinase
BCR-ABL	Breakpoint Cluster Region–Abelson Murine Leukemia Viral Oncogene Fusion Protein
BCR	Breakpoint Cluster Region
CML	Chronic Myeloid Leukemia
DCM	Dilated Cardiomyopathy
FDR	False Discovery Rate
MeSH	Medical Subject Headings
PPI	Protein–Protein Interaction
PCA	Principal Component Analysis
TAS	Transcriptional Activity Score
TKI	Tyrosine kinase inhibitor
